# Compounding Impacts of Human-Induced Water Stress and Climate Change on Water Availability

**DOI:** 10.1038/s41598-017-06765-0

**Published:** 2017-07-24

**Authors:** Ali Mehran, Amir AghaKouchak, Navid Nakhjiri, Michael J. Stewardson, Murray C. Peel, Thomas J. Phillips, Yoshihide Wada, Jakin K. Ravalico

**Affiliations:** 1 Department of Civil and Environmental Engineering, University of California, Irvine, CA 92697 USA; 2 Department of Infrastructure Engineering, The University of Melbourne, Parkville 3010 Victoria, Australia; 3 Program for Climate Model Diagnosis and Intercomparison, Lawrence Livermore National Laboratory, 7000 East Avenue, Livermore, CA 94550 USA; 4 NASA Goddard Institute for Space Studies, 2880 Broadway, New York, NY 10025 USA; 5 Center for Climate Systems Research, Columbia University, New York, USA; 6 Department of Physical Geography, Utrecht University, Utrecht, The Netherlands; 7 International Institute for Applied Systems Analysis, Laxenburg, Austria; 8 Melbourne Water, 990 La Trobe Street, Docklands, Victoria, 3008 Australia

## Abstract

The terrestrial phase of the water cycle can be seriously impacted by water management and human water use behavior (e.g., reservoir operation, and irrigation withdrawals). Here we outline a method for assessing water availability in a changing climate, while explicitly considering anthropogenic water demand scenarios and water supply infrastructure designed to cope with climatic extremes. The framework brings a top-down and bottom-up approach to provide localized water assessment based on local water supply infrastructure and projected water demands. When our framework is applied to southeastern Australia we find that, for some combinations of climatic change and water demand, the region could experience water stress similar or worse than the epic Millennium Drought. We show considering only the influence of future climate on water supply, and neglecting future changes in water demand and water storage augmentation might lead to opposing perspectives on future water availability. While human water use can significantly exacerbate climate change impacts on water availability, if managed well, it allows societies to react and adapt to a changing climate. The methodology we present offers a unique avenue for linking climatic and hydrologic processes to water resource supply and demand management and other human interactions.

## Introduction

**Figure 1:**
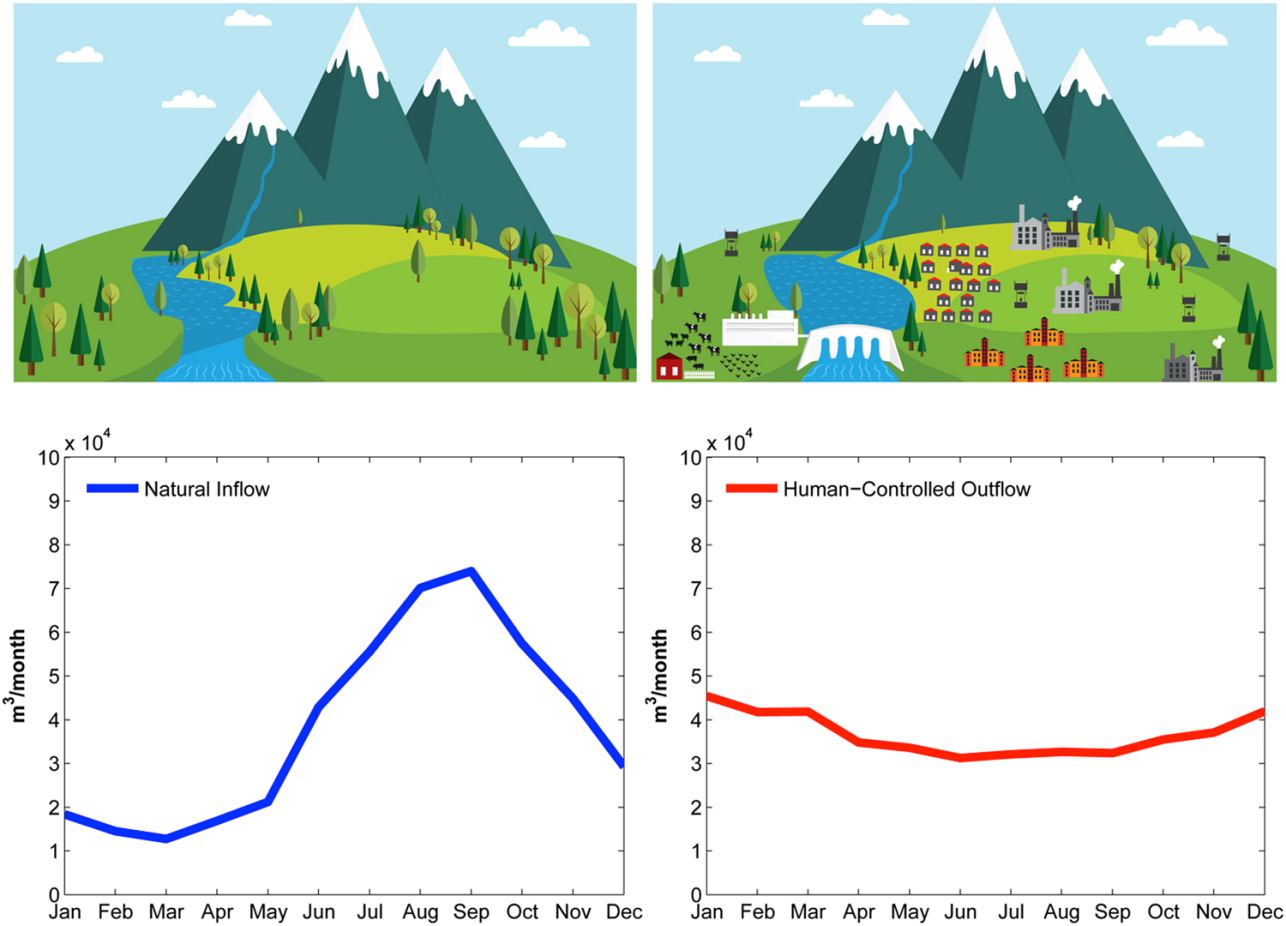
Anthropogenic activities alter the natural water cycle and distribution. The bottom row shows the mean monthly inflow to and outflow from Melbourne major reservoirs: (left) Natural stream flow upstream of the reservoirs before management by man-made infrastructure, (right) human-dominated outflow from the reservoirs.

Water resources are sensitive to climate change and variability^1–5^, especially in arid and semi-arid regions^6–8^. Regional and global hydrologic models forced with Global climate model simulations have been widely used to assess future changes in water resources^9–11^. Water availability is also closely associated with operations of water supply infrastructure (surface water reservoirs and desalination plants, etc.), and human water use behavior (e.g., growth and seasonal cycles in water demands)^12^. Some modeling frameworks used for climate/hydrology projections typically simulate the natural hydrologic cycle^13–17^ (Fig. 1 (top right)) without considering anthropogenic water demand, human interactions^18, 19^ and man-made infrastructure such as dams and reservoirs^20^ (Fig. 1(top left)). Storage infrastructure can significantly alter water flow and distribution^21^. Man-made surface reservoirs control^22^ about 20% of the global annual river discharge (~8000 km^3^ out of 40000 km^3^; ref. 23) and provide resilience against droughts, in addition to their role in water resource management and energy production^24–27^. Since early 2000s, several major modeling efforts have tackled integrating water demand, irrigation and other human dimensions in water stress and availability analysis^10, 28–41^.

Man-made local water supply infrastructure (in particular surface water reservoirs) affects future water availability because it is, generally speaking, built specifically to cope with climatic extremes. A system with distributed and different water storage, and therefore more local resilience, will be less vulnerable to climatic change and variability compared to a system with limited local capacity to cope with extremes. As a result, different regions will see different water availability changes depending on their local infrastructure and capacity to cope with variability or adapt to change. Omitting surface water reservoirs from large-scale water cycle models introduces a large source of uncertainty in current assessments of the global water cycle and hinders evaluation of climate change and variability on hydropower energy production^42^. Continental-scale closure errors of the water budget range from 13% (Europe) to 21% (Australia)^43^, which can be attributed to input data uncertainty, modeling assumptions and anthropogenic influences on water distribution. For this reason, hydrologic models should include an explicit numerical description of the water balance dynamics of reservoirs (surface and subsurface) and other large water bodies^11, 44, 45^.

Over the past century, substantial growth in population, industrial and agricultural activities, and living standards (i.e. per capita water use) have exacerbated water stress in many parts of the world^46, 47^, especially in semi-arid and arid regions. In fact, even if future water supplies remain unchanged, societies should be prepared for more competition over water due to ever-increasing anthropogenic water demand. A*nthropogenic drought* ^46^ is inevitable if increasing demand, dominated by human water use, exceeds water availability. Change in human water demands is another component that is often ignored in assessing future climatic impacts on water resources^48–50^.

We focus on the Melbourne metropolitan area in the southeast of Australia where most of the water for consumptive or industrial use comes from large reservoirs in protected areas (Figure S1). In this area reservoirs fundamentally change the distribution of water availability throughout the year to meet local human, industrial, agricultural and environmental water demand (Fig. 1). Most of the natural inflow occurs during July to October when water demand is relatively low. The water stored during this wet season is released in the summer when demand significantly exceeds inflow rates (compare the natural flow with the outflow of man-made reservoirs in Fig. 1). Thus, an accurate assessment of climate change impacts on water resources availability in this region requires explicit consideration of the dynamic human interactions^19^. During the past century, Melbourne has suffered several major water crises and severe droughts. The most extreme was the well-known Millennium Drought (1997–2009)^51–53^, which drained the reservoirs and caused major wildfires with significant economic and human losses^51, 54–56^. The water supply catchments for metropolitan area of Melbourne are a quintessential example of a highly-regulated water system, with a number of reservoirs that enhance local resilience and help the region cope with climatic extremes through water storage and redistribution (though the area is still vulnerable to climate change and variability)^51^.

While previous studies have addressed integrating human interactions in earth system or hydrologic models, there are still major modeling challenges. Previous studies do not include local reservoir model calibration based on water storage information, which is closely associated with local resilience to extreme events. Furthermore, global water demand projections used for assessing human influence do not include local policies and management practices. This paper outlines a nested modeling framework that explicitly accounts for future water demand and man-made infrastructure such as reservoirs and high reliability alternative water sources (local resilience) when evaluating the impact of climate change on water resources (see Methods Section). The model is designed to reproduce historical observations and allows for integrating multiple types of infrastructure (e.g., reservoirs and other high reliability alternative water sources). The proposed nested framework allows tailoring the model to local conditions and including bottom-up information such as future demand scenarios. In this study, human water demand provided local information based on different population and growth policies.

We assess future climate change impacts on water resources in Melbourne Metropolitan area using climate change projections from 12 global models participating in the fifth phase of the Coupled Model Intercomparison Project (CMIP5). Each model is subjected to a scenario of prescribed exponentially growing 21^st^ century greenhouse gas (GHG) emissions or concentrations—the Representative Concentration Pathways 8.5 scenario (RCP8.5, see ref. 50 and Table S1 in Supplementary Materials). We account for human influence by integrating man-made reservoirs (Figure S1) and by considering 17 different future water demand scenarios ranging from very optimistic to very unfavorable (see Methods Section and Table S2). These demand scenarios weight the effects of different assumptions of population, industrial and agricultural growth, and consumption behaviors. Our explicit consideration of water demand scenarios, involving a localized bottom-up accounting for human influence and local conditions, is a major advance from the conventional large-scale, top-down approach^47, 57, 58^.

Our analysis proceeds as follows: we first define and set up a water balance model of the major reservoirs^44, 45^ in the Melbourne area (Maroondah, O’Shannassy, Upper Yarra, and Thomson – Figure S1), and calibrate this reservoir model using a historical record of inflow and water use data (Methods Section). Then, a distributed hydrologic model is used to obtain future inflow to the major reservoirs based on projections from the CMIP5 precipitation and temperature simulations. Projected water demand as well as water available from a local desalination plant are used to assess water stress in the projection period (2020–2035) relative to the baseline (1995–2010).

**Figure 2:**
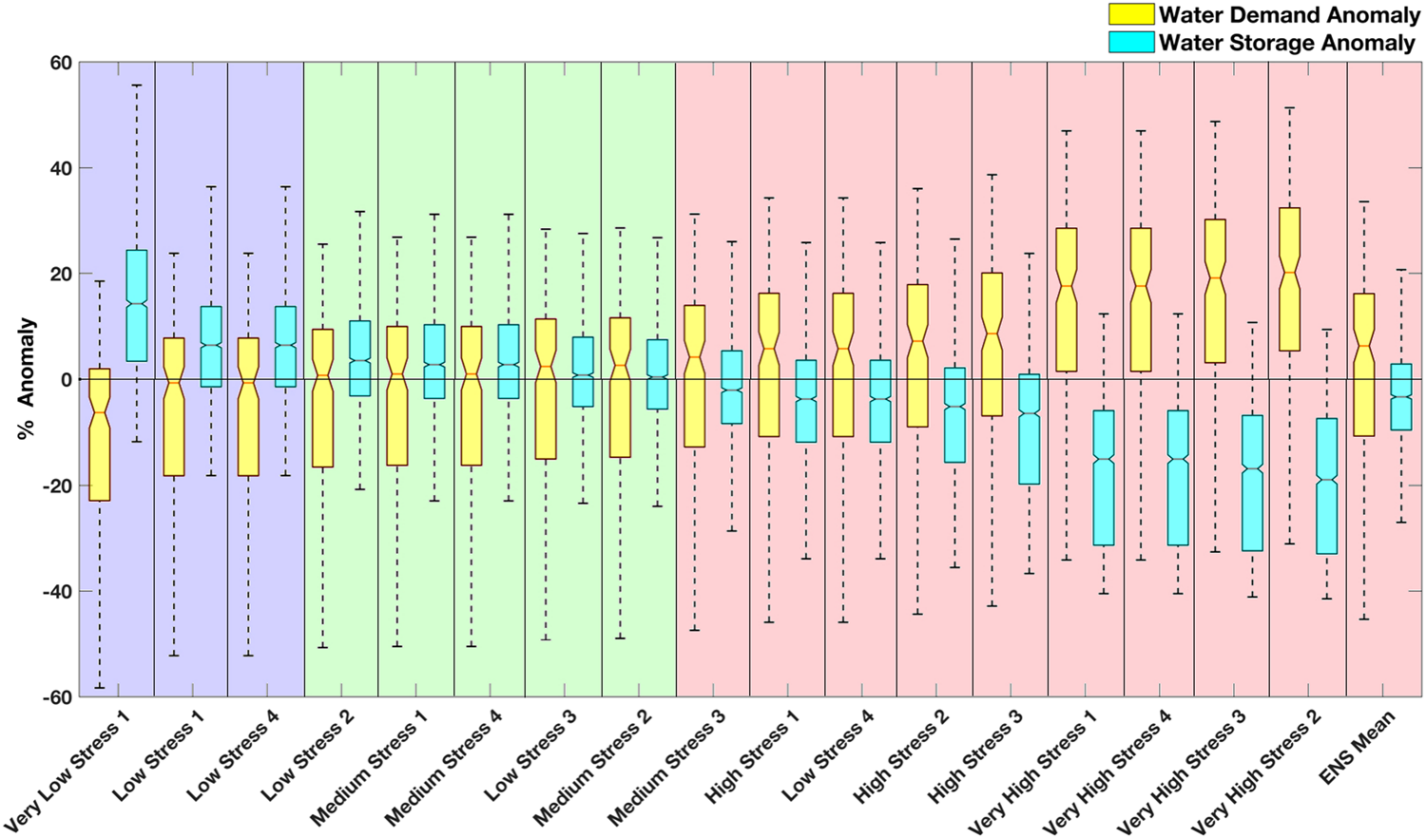
Melbourne future water demand scenarios (see Table S2) and their corresponding projected reservoir water storage anomalies in 2020–2035 relative to the baseline (1995–2010). The blue-shaded boxplots indicate that optimistic future water demand scenarios (demand in the projection period would be less than the baseline), leading to more water storage in the projection period relative to the baseline. The green-shaded boxplots show scenarios in which future demand is more than the baseline, but the projected average storage anomalies still remain positive (i.e., despite increases in the future demand, because of the storage infrastructure, the system would not experience water stress worse than the baseline period which includes the Millennium Drought). The red-shaded boxplots exhibit scenarios that the future demand significantly exceeds that of the baseline and the projected average storage anomalies are negative under the RCP 8.5 climate projections (i.e., with the current storage capacity, considering both climatic change and future demand, the region would experience more water stress in the future relative to the baseline period).

Melbourne’s future water availability, considering the available storage infrastructure, projected climate, and all expected future demand scenarios (Table S2) are summarized in Fig. 2. The purple-shaded region (far left) shows optimistic future water demand scenarios in which the demand in the projection period (2020–2035) is less than the baseline (1995–2010). These scenarios lead to more mean water storage in the projection period relative to the baseline (i.e., positive mean storage anomalies or more available water relative to the baseline). The green-shaded region shows scenarios in which future demand is greater than the baseline, but the projected average storage anomalies still remain positive. That is, despite increases in the future demand, the system would not experience water stress worse than that of the baseline period (which includes the Millennium Drought). The red-shaded region corresponds to scenarios in which future demand significantly exceeds that of the baseline, and projected average storage anomalies are negative under the RCP8.5 climate projections. This latter indicates that the Melbourne area would experience more water stress in the future relative to the baseline period, given the current storage capacity and considering both climatic change and future demand.

Considering only the future climate and ignoring both future demand and storage capacity leads to a different perspective on future water availability (Figure S2 in Supplementary Materials). Model simulations of the future indicate that under RCP 8.5, the region will experience significant reduction in runoff during 2020–2035 relative to 1995–2010, and the ensemble mean of all future inflow simulations is negative (i.e., indicates more water stress in the future relative to the baseline). The inflow, alone, is not a good indicator of water availability since: (a) it does not include the amount of water needed for human consumption; or (b) how much of the inflow can be stored in reservoirs or augmented by other infrastructure. Figure 2 offers a unique perspective that involves both of these issues in future water availability assessment (compare Fig. 2 with Figure S2). In fact, we argue that without accounting for water storage and human water needs, estimates of future water availability (or stress) may not be reliable.

While 14 scenarios project higher water demands in the future relative to that of the baseline period, only 9 of those scenarios (Very Low Stress to Very High Stress in Table S2) lead to storage deficit more extreme than the baseline period under the RCP8.5 climate change assumption (Fig. 2). Note that the baseline period includes the Millennium drought. These results suggest that the combination of climatic change and several projected water demand scenarios (including the ensemble mean of the selected climate models and demand scenarios) would likely lead to water stress conditions more extreme than the Millennium drought. Furthermore, with the available storage infrastructure, if the demand is restricted to the low or medium demand scenarios (blue and green regions in Fig. 2), the net average storage remains above the baseline period. Comparing low (blue) and high (red) water demand scenarios in Fig. 2 highlights that human-induced water stress significantly exacerbates climate impacts on water availability. While human water use can cause or intensify water stress, if managed well, it allows societies to react and adapt to the projected conditions (blue and green regions in Fig. 2).

**Figure 3:**
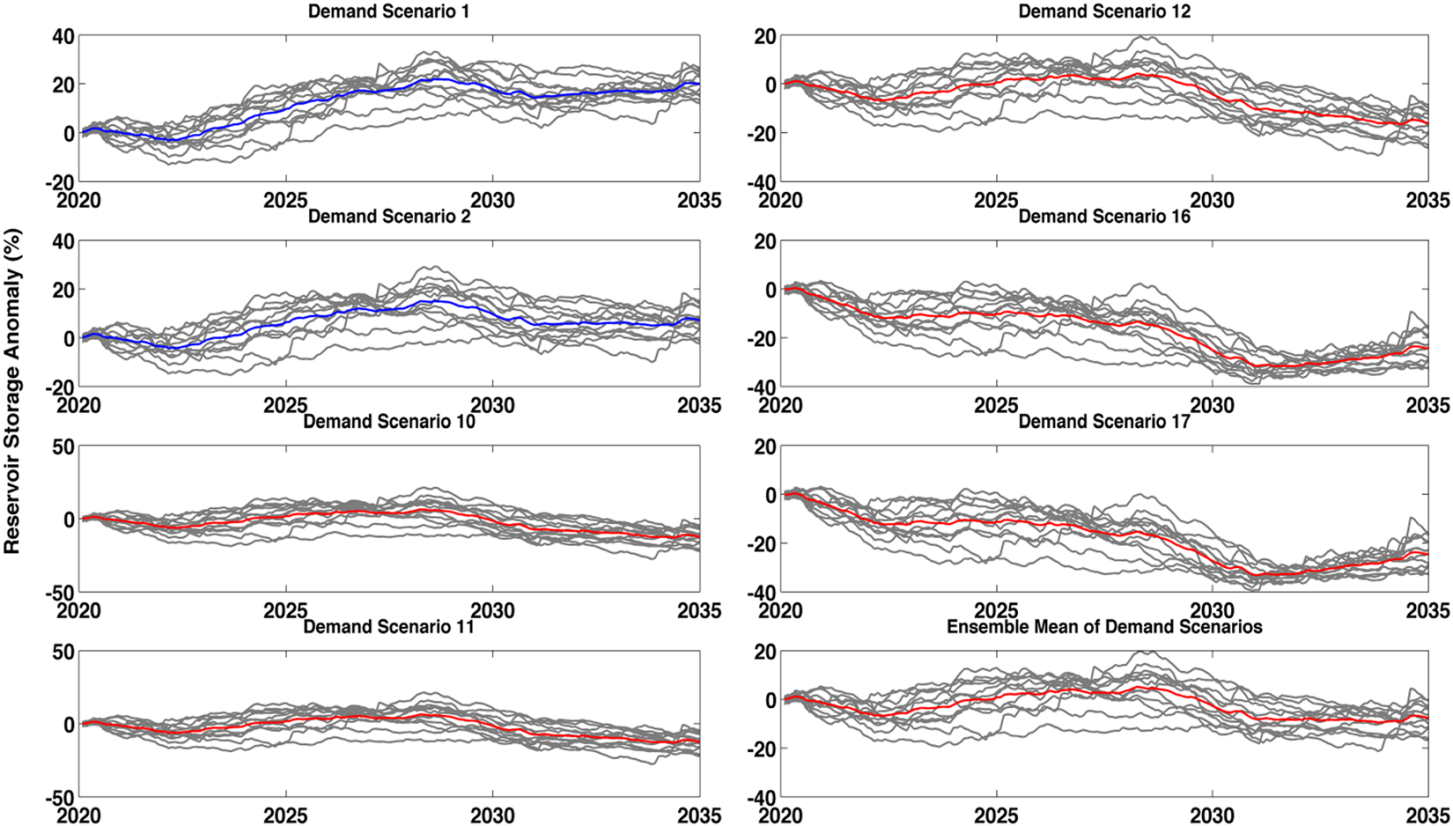
Reservoir water storage anomalies considering future climate and projected demand in 2020–2035 relative to the baseline (1995–2010). Each gray line is a model output driven by one single climate model. A net positive ensemble average (blue) indicates that on average the future storage will be more than the baseline, whereas a negative storage (red) indicates that the system will expect more water stress relative to the baseline (i.e., Millennium Drought) – for demand scenarios see Table S2 in Supplementary Materials.

The framework presented in the Methods Section offers time series of change in storage based on the future climate and water demand scenarios. For selected water demand discussed in Table S2, Fig. 3 displays time series of reservoir storage anomalies (%). The gray lines represent future projections of different climate models relative to the baseline, whereas the red and blue lines denote the ensemble means. The ensemble means that lead to an overall positive or negative anomaly are shown in blue, and red, respectively. In the ensemble means marked by blue, storage anomalies in the reservoirs remain positive during the projection period relative to the baseline period. Yet, for most other demand scenarios the ensemble means are negative (red lines) and the storage of the reservoirs would be below the historical baseline level. It is worth mentioning that uncertainties in the future model projections and demand scenarios are substantial (see Figs 2 and S2) and the variability should be considered along with the mean behavior of the system. We also acknowledge that accounting for climate change impacts on water supply (e.g., input to reservoirs) does not fully capture the spread or uncertainty in the water storage scenarios.

**Figure 4:**
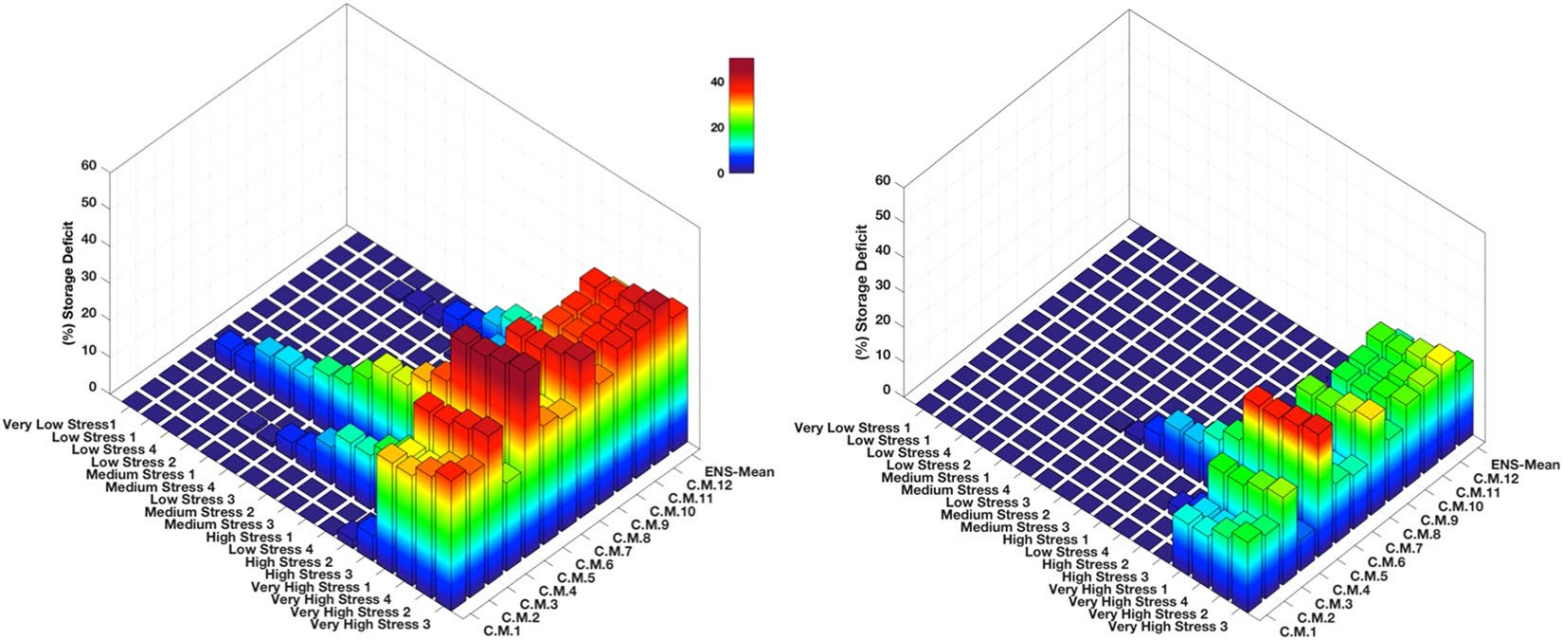
(**a**) Melbourne’s average water storage deficit based on different climate model simulations (C.M.1–12–Table S1) and their ensemble mean (ENS-Mean) under different future demand scenarios (Table S2) in 2020–2035 relative to the baseline (1995–2010). (**b**) Same as (**a**) but considering alternative water sources with the annual capacity of 150 GL.

This modeling framework aims to show whether the current reservoirs provide sufficient local resilience against the projected climatic change and increase in water demand. In scenarios that lead to negative storage anomalies, the available storage capacity may not be sufficient to buffer against future climate change and water demand increases^51^. The proposed modeling framework allows managers to assess climate change impact on water resources while including water supply infrastructure such as desalination plants. Figure 4 shows to what extent a high reliability alternative water source of 150 GL per year can buffer water shortages in a changing climate and expected future demand scenarios. For all combinations of the climate model simulations for the future (Table S1) and projected water demand (Table S2), Fig. 4 shows the storage deficit with and without the addition of alternative water sources of 150 GL per year. The addition of a high reliability alternative water source reduces the storage deficit substantially (compare Fig. 4a and b). However, under some combinations of future water demand and projected climate change (RCP 8.5), the region will likely experience water stress conditions more extreme than the Millennium drought, despite the available water storage and supply infrastructure.

In recent years, assessing future changes in water availability has received a great deal of attention. Increased human water use, which will likely increase in the future, is recognized as an important component of local water stress^33, 37, 39, 59–62^. Our results suggest that predictions of future water availability should consider not only the future climate, but also future localized water demand and water supply infrastructure to cope with climate variability. In fact, climate alone is not a very good predictor of future water scarcity, because of the many complex ways in which humans acquire and use water. Here, we present a case that isolates the interaction between just three components (projected climate change, projected human water demand, and local storage) of this much more complex system. Indeed, for the same set of climate scenarios, existing water supply infrastructure is either adequate or not depending on projections of water demand. The interaction between human and climate can dramatically enhance or reduce local vulnerability to water stress. A question often ignored in the climate community is: To what extent will uncontrolled growth with concomitant increase in water use exacerbate future water stress? Our framework offers a unique way to incorporate the anthropogenic water demand (human-induced water stress) and local capacity to cope with extremes when assessing future climate. This approach can be used to assess unexpected consequences that can occur when both demand and climate vary within normal bands, but their combination leads occasionally to acute water scarcity.

## Methods

Future simulations of daily precipitation and temperature from the Coupled Model Intercomparison Project Phase 5 (CMIP5; ref. 63) are used to estimate future water availability. The climate model simulations are summarized in Table S1 (Supplementary Materials). CMIP5 includes a suite of historical and future climate simulations that are subjected to common GHG emissions or concentration scenarios, as reported in the fifth assessment report of the Intergovernmental Panel on Climate Change (IPCC, 2013). We chose CMIP5 simulations of the RCP 8.5 scenario of exponentially growing 21^st^ century CO_2_ emissions or concentrations. In addition, Melbourne water (Table S2 in Supplementary Materials) supplied a wide range of hypothetical water demand scenarios, from very low stress to very high stress. These data are estimated from different projections of population growth, industrial and agricultural development, and consumption behavior. The observed inflow and outflow to Melbourne major reservoirs (Maroondah, O’Shannassy, Upper Yarra, and Thomson) are from Melbourne Water, and are used for model calibration.

Estimates of local surface runoff (e.g., Figure S2) are derived from the spatially distributed PCR-GLOBWB model^44^, a process-based conceptual hydrologic model that includes a surface water and groundwater component. This model has been used extensively in previous studies^44, 45, 64^. The PCR-GLOBWB model is forced with daily CMIP5 precipitation and temperature simulations after bias adjustment^65^ to generate inflow to the reservoirs (see Figure S2) and reservoir storage (Figure S3) based on the projected demand (Table S2). A reservoir model is then nested with the hydrologic model and used to estimate the water storage^42, 44, 66, 67^. The storage, *S* (L^3^) of the reservoir is computed using a simple water balance equation

1 $$\frac{\partial S}{\partial t}={Q}_{{\rm{in}}}-{Q}_{{\rm{out}}}-{Q}_{{\rm{add}}}-{Q}_{{\rm{evap}}},$$

where *t* (T) denotes time, *Q*_in_ (L^3^T^−1^) and *Q*_out_ (L^3^T^−1^) are the reservoir inflow and outflow volume rate, respectively, *Q*_add_ (L^3^T^−1^) defines the additional release from the reservoir for flood control and reservoir management, and *Q*_evap_ (L^3^T^−1^) signifies evaporation. All water balance terms are non-negative and to simplify their notation, we omit dependence on time. Equation (1) is solved numerically using a fixed monthly integration time step using values of the initial and maximum reservoir storage, *S*_0_ and *S*_max_, respectively, and monthly inflow volume rates, *Q*_in_ and demand data, *D* (L^3^T^−1^) from Melbourne Water. The reservoir outflow rate, *Q*_out_ is dependent on demand, the actual water storage in the reservoir, and the long-term mean reservoir inflow rate, ${\bar{Q}}_{{\rm{in}}}$ (L^3^T^−1^) using

2 $${Q}_{{\rm{out}}}=\,\max (\min ({Q}_{d},\propto S),g(S){\bar{Q}}_{{\rm{in}}}),$$

where *Q*_d_ (L^3^T^−1^) denotes the actual reservoir outflow (or withdrawal) rate required to satisfy the monthly demand, α (T^−1^) is a nuisance variable with value unity used to resolve the unit mismatch between volume and rate, and the function *g*(·) calculates the so-called potential release factor. This potential release factor specifies the portion of inflow that is allowed to be released depending on the storage. The variable *Q*_d_ is computed as follows

3 $${Q}_{{\rm{d}}}=\{\begin{array}{c}(\frac{S\,}{{S}_{{\rm{low}}}})D\,{\rm{if}}\,S\,\le \,{S}_{{\rm{\min }}}\\ D\qquad \quad {\rm{if}}\,S > {S}_{{\rm{\min }}}\end{array},$$

where *S*_low_ (L^3^) is the (unknown) minimum storage of the reservoir required to satisfy the water demand. Thus, demand will be met pending sufficient storage otherwise the release from the reservoir is reduced to secure future water availability. The potential release factor, *g*(*·*) ∈ [0, 1], is unitless and dependent only on the actual storage in the reservoir

4 $$g(S)=\{\begin{array}{l}0\phantom{\rule{4em}{0ex}}\,{\rm{if}}\,S\le {S}_{{\rm{low}}}\\ \frac{S-{S}_{{\rm{low}}}}{{S}_{{\rm{up}}}-{S}_{{\rm{low}}}}\,{\rm{if}}\,{S}_{{\rm{low}}} < S < {S}_{{\rm{up}}}\\ \,1\phantom{\rule{4em}{0ex}}{\rm{if}}\,S\ge {S}_{{\rm{up}}},\end{array}$$

where *S*_up_ (L) is the (unknown) lowest reservoir storage required to operate at full capacity. This variable is not to be confused with *S*_max_. The values of *S*_low_ and *S*_up_ need to be carefully determined and are dependent on (among others) the (geologic, hydraulic) properties of the reservoir, size of the contributing area, climatic conditions, operational demand, and management practice.

The third term (*Q*_add_) of the water balance in Equation 1 is determined by reservoir operation. The management of the reservoir should be tailored specifically to guarantee continued water availability for industry and the public, guarantee ecosystem sustainability and protecting simultaneously surrounding areas against flooding. The following equation is used to calculate *Q*_add_

5 $${Q}_{{\rm{add}}}=(\frac{S-{S}_{{\rm{up}}}}{{{\rm{S}}}_{{\rm{\max }}}-{S}_{{\rm{up}}}})({Q}_{{\rm{b}}}-{Q}_{{\rm{out}}}),$$

where *Q*_b_ (LT^−1^) is the river bank-full discharge (i.e., maximum attainable reservoir inflow rate) and computed using ${Q}_{{\rm{b}}}={\beta }_{{\rm{\max }}}{\bar{Q}}_{{\rm{in}}}$ where *β*_max_ is a unitless rating coefficient. In case the storage of the reservoir exceeds the maximum storage, *S*_max_, then the first term (between brackets) at the right hand side is set to unity, and the excess water, *S* − *S*_max_ released immediately from the reservoir.

Finally, the last term of Equation 1, reservoir evaporation, is computed from the reservoir storage using

6 $${Q}_{{\rm{evap}}}={\rm{\gamma }}S,$$

where γ (T^−1^) is a unitless evaporation coefficient dependent on climatic conditions and the surface area of the reservoir.

Our initial simulations have shown that *Q*_add_ does not play a significant role in the long-term storage estimates of the reservoir, but only contributes to the reservoir outflow during very wet months (flood control). What is more, reservoir evaporation is negligible small. Thus, the reservoir model and storage is primarily dominated by the first two terms, *Q*_in_ and *Q*_out_ of Equation 1.

We use herein a 15-year historical record (1995–2010) of monthly demand data (*D*), and reservoir inflow (*Q*_in_) and outflow (*Q*_out_) rates of the Melbourne area. As the available demand data does not distinguish among the four main contributing reservoirs (Maroondah, O’Shannassy, Upper Yarra, and Thomson) we simulate their combined storage with Equation 1 using cumulative values of their inflow rates. The initial and maximum reservoir storage are set to *S*_0_ = 1,490 Mm^3^ (1/31/1995) and *S*_max_ = 2,000 Mm^3^, respectively, the value of ${\beta }_{{\rm{\max }}}$ is set equal to 2.3 (−), *γ* = 0.0015 (month^−1^) and ${\bar{Q}}_{{\rm{in}}}$ = 37.136 Mm^3^ month^−1^. These values are consistent with field observations and actual data. The values of *S*_low_ and *S*_up_ are determined by reservoir management, and assumed to vary dynamically per month. Their 24-values are estimated using Bayesian inference with the DREAM algorithm^68–70^ using historical measurements of the cumulative monthly storage.

The DREAM algorithm is a Markov chain Monte Carlo (MCMC) simulation algorithm that returns the optimum values of the reservoir parameters. In short, in DREAM, *N* different Markov chains are run simultaneously in parallel. If the state of a single chain is given by the *d = *24 dimensional vector **x** with values of *S*_low_ and *S*_up_, then at each generation *i* the *N* chains in DREAM define a population **X**_*i*_ which corresponds to an *N × d* matrix, with each chain as a row. Multivariate proposals are generated on the fly from the collection of chains, ***X***_*i*_ using differential evolution^71, 72^. By accepting each proposal with Metropolis probability a Markov chain is obtained, the stationary or limiting distribution of which is the posterior distribution (see the proof in refs 68, 69 and 73). If the initial population is drawn from the prior distribution, then DREAM translates this sample into a posterior population. We assumed a uniform prior distribution of the 24 parameters, whereas a classical least squares type likelihood function was used to summarize the distance between the observed and simulated monthly storage volumes. The first ten years of the 17-year data record (1995–2004) were used for posterior inference of the monthly *S*_low_ and *S*_up_ values, whereas the remaining 7-year record (2005–2011) is used for model evaluation purposes. Table S3 lists the estimated parameters and their standard deviations. Table S4 summarizes the model efficiency coefficients for the calibration and evaluation periods. As shown in Table S4 and Figure S3, the simulated storage of the reservoir model closely tracks the observed storage data.

## Electronic supplementary material

Supplementary Information
