# Supplementary material for: Compounding Impacts of Human-Induced Water Stress and Climate Change on Water Availability
Source: Sci Rep. 2017 Jul 24;7:6282. doi: 10.1038/s41598-017-06765-0 (PMC5524826; doi:10.1038/s41598-017-06765-0)
Supplement: Supplementary file 1 — Supplementary Information [file 41598_2017_6765_MOESM1_ESM.pdf]

Compounding Impacts of Human-Induced Water Stress and Climate Change on Water Availability

Ali Mehran[1], Amir AghaKouchak[1], Navid Nakhjiri[1], Michael J. Stewardson[2], Murray C. Peel[2], Thomas J. Phillips[3], Yoshihide Wada[4,5,6,7], Jakin K. Ravalico[8]

[1]Department of Civil and Environmental Engineering, University of California, Irvine, CA 92697, USA

[2]Department of Infrastructure Engineering, The University of Melbourne, Parkville 3010, Victoria, Australia.

[3] Program for Climate Model Diagnosis and Intercomparison, Lawrence Livermore National Laboratory, 7000 East Avenue, Livermore, CA 94550, USA.

[4]NASA Goddard Institute for Space Studies, 2880 Broadway, New York, NY 10025 USA.

[5]Center for Climate Systems Research, Columbia University, New York, USA

[6]Department of Physical Geography, Utrecht University, Utrecht, The Netherlands

[7]International Institute for Applied Systems Analysis, Laxenburg, Austria

[8]Melbourne Water, 990 La Trobe Street, Docklands, Victoria 3008, Australia

Correspondence and requests for materials should be addressed to A.M. (email: amehran@ucla.edu)

Supplementary Materials

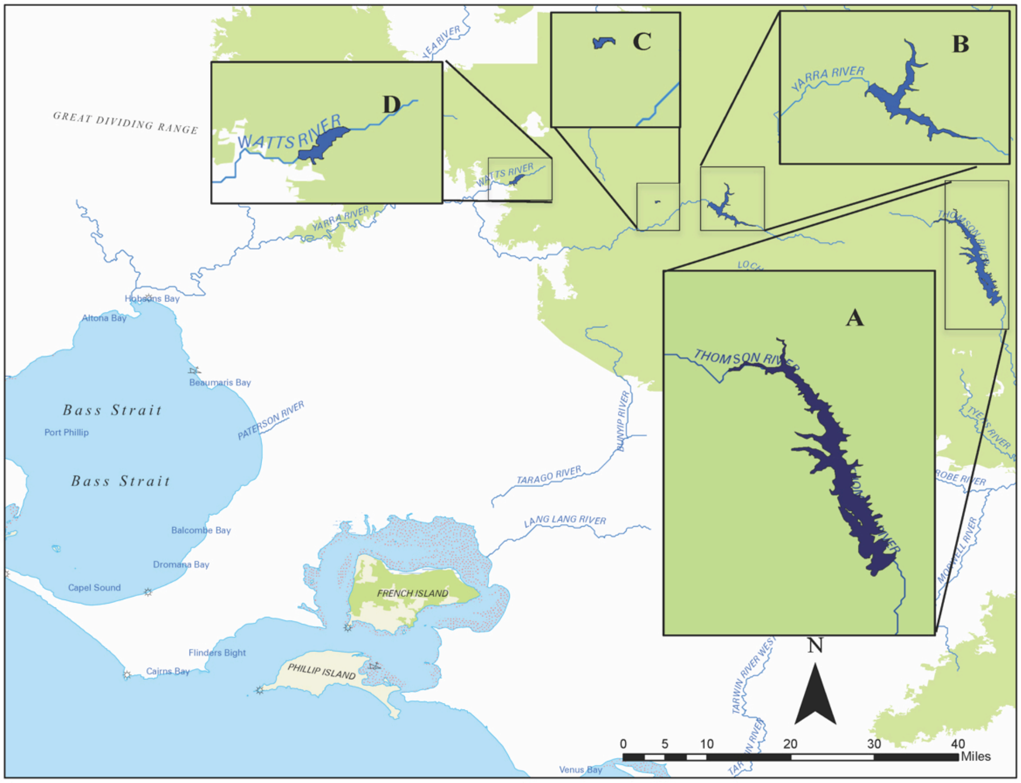

Figure S1 – Melbourne major reservoirs including Thomson (A), Upper Yarra (B), O'Shannassy (C), and Maroondah (D) (this figure was created by ArcGIS 10.3, http://desktop.arcgis.com/en/).

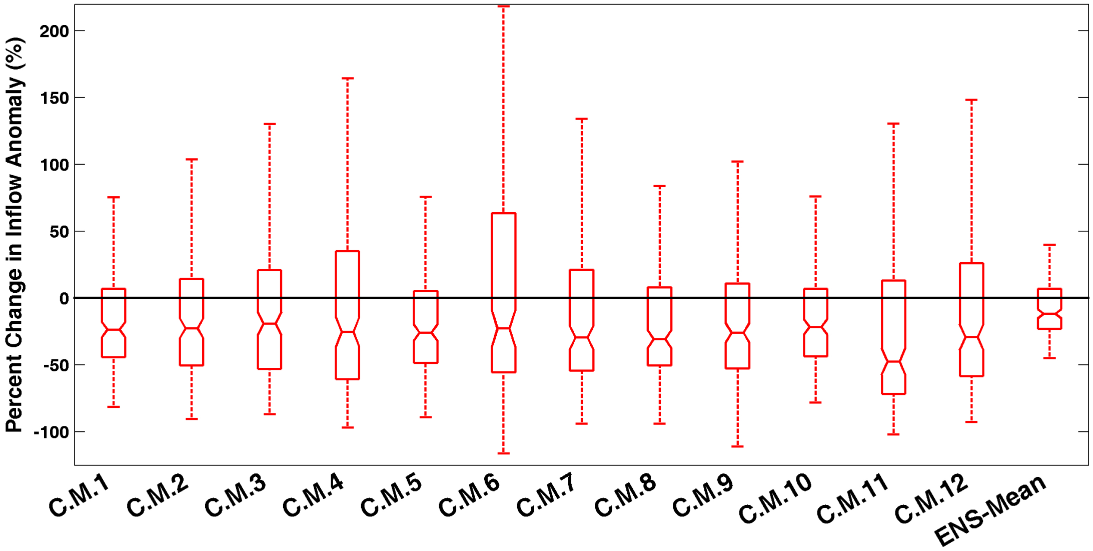

Figure S2 – Projected runoff anomalies in 2020-2035 relative to the baseline (1995-2010) based on PCR-GLOBWB (Methods Section) forced by CMIP5 RCP 8.5 climate model simulations listed in Table S1 and their ensemble mean (ENS-Mean). Each boxplot shows monthly variations of the future projections relative to those of historical simulations (i.e., the ranges of boxplots show monthly climate variability represented by each climate model, and not model uncertainty). The ensemble means of inflows for all models are negative indicating more water stress in the projection period relative to the baseline.

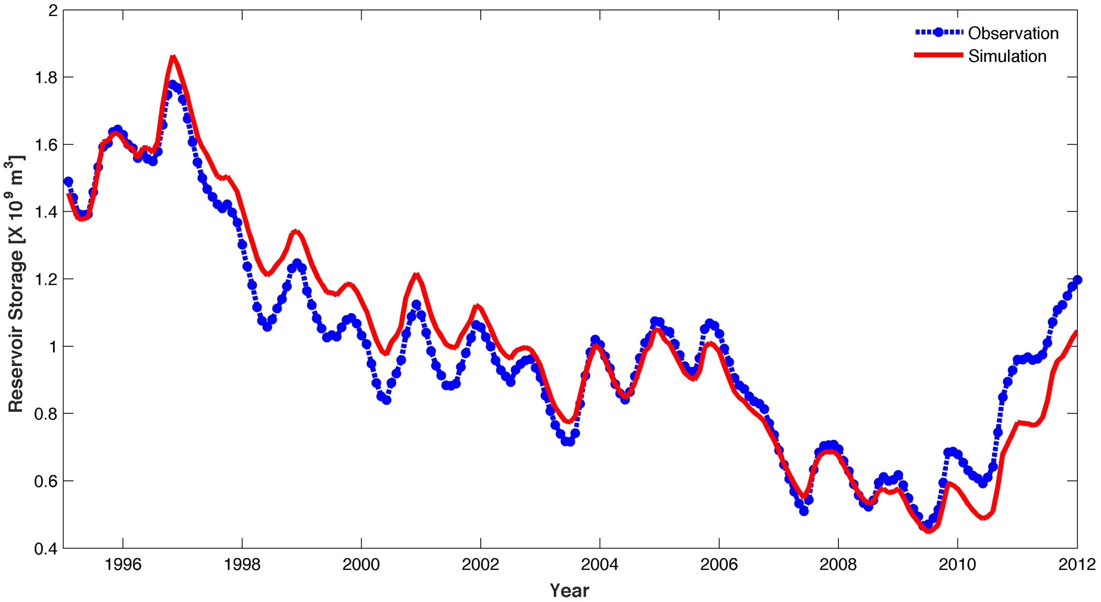

Figure S3 – Water storage observation compared with reservoir model simulation during baseline period (1995-2010), statistics are represented in Table S4.

Table S1 – List of the CMIP5 model simulations used for simulating the RCP8.5 future-climate scenario*.

CMIP5 Climate Models		Institution
**1**	BNU_ESM	College of Global Change and Earth System Science, Beijing Normal University
**2**	CCSM4	National Center for Atmospheric Research (NCAR), USA
**3**	CESM1_BGC	National Science Foundation, Department of Energy, and NCAR, USA
**4**	FIO_ESM	The First Institute of Oceanography, China
**5**	MIROC_ESM	Japan Agency for Marine-Earth Science and Technology, Atmosphere and Ocean Research Institute (The University of Tokyo), and National Institute for Environmental Studies, Japan
**6**	MPI_ESM_LR	Max Planck Institute for Meteorology (MPI-M), Germany
**7**	MRI_CGCM3	Meteorological Research Institute, Japan
**8**	NorESM1_M	Norwegian Climate Centre, Norway
**9**	IPSL_CM5a_LR	Institut Pierre-Simon Laplace, France
**10**	GFDL_ESM2M	National Oceanic and Atmospheric Administration (NOAA) Geophysical Fluid Dynamics Laboratory, USA
**11**	BCC_CSM1	Beijing Climate Center, China
**12**	INMCM4_ESM	Institute for Numerical Mathematics, Russia

*Note, Earth System Models (denoted by the suffix 'ESM') predict temporally varying stores of carbon in land, ocean, and atmospheric components of the climate system. The ESMs accept prescribed values of RCP8.5-scenario *emissions* (fluxes) of greenhouse gases (GHGs) as inputs, and then predict atmospheric greenhouse-gas *concentrations* (e.g. in parts-per-million units) as well as the associated radiative forcings and many other features of global climate. Conventional climate models (e.g. CCSM4, MRI_CGCM3, etc.) do not predict the global carbon cycle, but instead accept RCP8.5-prescribed GHG *concentrations* as inputs, and then predict the associated radiative forcings and other global climatic features.

Table S2 – Statistics of future water demand (GL/yr - Gigaliters per year) scenarios available from Melbourne Water for 2020-2035. Projected demands are based on future climate conditions, population, energy price, manufacturing needs, liveability, domestic needs, and bushfire (BF) trends.

	Demand Scenario	Mean	Range (Max-Min)
**1**	Very Low Stress 1	404.8	29.9
**2**	Low Stress 1	429.4	36.7
**3**	Low Stress 4	429.4	36.7
**4**	Low Stress 2	436.6	38.9
**5**	Medium Stress 1	439.2	45.5
**6**	Medium Stress 4	439.2	45.5
**7**	Low Stress 3	445.2	48.5
**8**	Medium Stress 2	446.4	47.7
**9**	Medium Stress 3	455.0	57.3
**10**	High Stress 1	463.6	67.1
**11**	Low Stress 4	463.6	67.1
**12**	High Stress 2	470.8	69.2
**13**	High Stress 3	479.4	78.8
**14**	Very High Stress 1	518.5	74.5
**15**	Very High Stress 4	518.5	74.5
**16**	Very High Stress 2	525.7	76.7
**17**	Very High Stress 3	534.3	86.2

Table S3 – Posterior mean and standard deviation of the model parameters.

**Parameters**	S_{low}		S_{up}	
**Month**	Mean	Standard Deviation	Mean	Standard Deviation
**Jan**	0.35	0.17	0.73	0.09
**Feb**	0.36	0.17	0.76	0.10
**Mar**	0.46	0.11	0.73	0.10
**Apr**	0.48	0.08	0.73	0.11
**May**	0.49	0.05	0.72	0.11
**Jun**	0.49	0.02	0.71	0.11
**Jul**	0.49	0.03	0.72	0.12
**Aug**	0.48	0.06	0.7	0.12
**Sep**	0.48	0.08	0.68	0.09
**Oct**	0.40	0.15	0.71	0.09
**Nov**	0.31	0.17	0.74	0.08
**Dec**	0.31	0.17	0.72	0.08

Table S4 – Model efficiency coefficients for the calibration and evaluation periods.

Summary Statistics	Calibration 1995-2004	Evaluation 2005-2011
Spearman Correlation	0.98	0.83
Correlation Coefficient	0.98	0.82
Nash Sutcliffe Coefficient	0.88	0.57
Relative Mean Error	3.35%	5.03%
